# Astounding Fibro Lipoma of Spermatic Cord: A Diagnostic Dilemma

**DOI:** 10.1155/2021/7139109

**Published:** 2021-09-04

**Authors:** Gupta Rohit, Chinniahnapalaya Pandurangaiah Hariprasad, Shreekant Bharti, Anil Kumar, Shiv Shankar Paswan, Surya Vikram, Atul Anand, Jijo Anto

**Affiliations:** ^1^Department of General Surgery, All India Institute of Medical Sciences, Patna, India; ^2^Department of Pathology and Lab Medicine, All India Institute of Medical Sciences, Patna, India

## Abstract

**Background:**

Fibro lipoma of the spermatic cord is a very rare tumor with few cases reported in literature. Atypical presentation and site of swelling mystifies the diagnosis and creates bewildering situation intraoperatively. *Case Summary*. A 30-year-old farmer presented with an elastic firm nonreducible, nontender swelling at inguinoscrotal region with positive cough impulse and history of laparoscopic inguinal hernia repair 3 years ago. Ultrasonography of the swelling revealed a heteroechoic lesion of size 7 × 6 centimeter with probable features of lipoma or desmoid. Fine needle aspiration drawn in consideration of the diagnostic dilemma reported a benign lipomatous swelling which on final histopathology turned out to be a fibro lipoma of size 7 × 6 × 5 cm.

**Conclusion:**

Considering the age and presentation of the patient, it was astonishing. It provided an insight to the occurrence of fibro lipoma even in younger age group which in fact is the first case of its kind as per best of our knowledge. Malignancy should be ruled out in such cases, and complete excision is the treatment of choice.

## 1. Introduction

Paratesticular tumor of primary origin accounts for only 7 to 10 percent of all intrascrotal tumor, of which majority arises from the spermatic cord [1]. Fibro lipoma of the spermatic cord is an extremely rare tumor especially in a young healthy individual. Ill-defined inguinoscrotal swellings with positive cough impulse possess a diagnostic dilemma to the treating surgeon. We present a rare case of left sided inguinoscrotal swelling in a patient previously operated for left sided inguinal hernia which to our utter surprise turned out to be a fibro lipoma of the spermatic cord. This case report is in line with SCARE criteria [2].

## 2. Case Report

**Figure 1:**
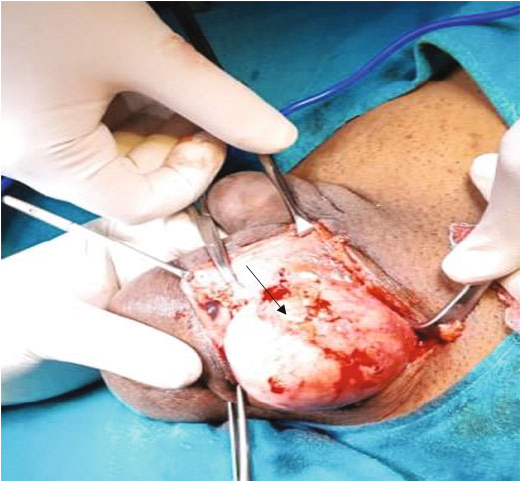
Intraoperative image showing firm grayish white well-encapsulated tumor originating from the spermatic cord (arrowhead).

**Figure 2:**
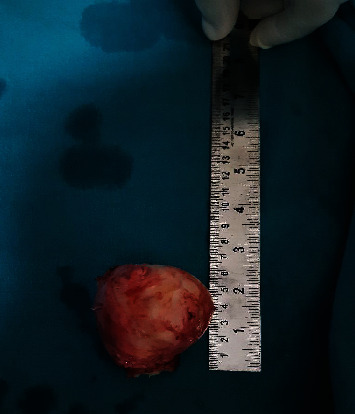
Firm grayish white tumor measuring 7 cm × 6 cm × 5 cm.

**Figure 3:**
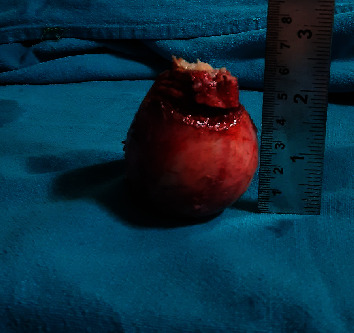
Excised pedunculated globular tumor of size 7 cm × 6 cm × 5 cm.

**Figure 4:**
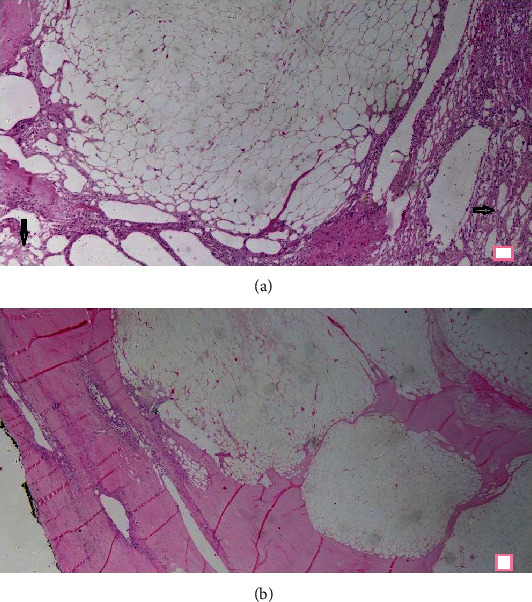
H&E, 20x magnification. Lobules of mature adipocytes present within the stroma of spermatic cord along with highlighted pampiniform plexuses (black arrows) (a). Lobules of mature fat forming a mass within the cord tissue, separated by fibrous septae of varying thicknesses (b).

A 30-year-old male, farmer by occupation, presented to the general surgery outpatient department of our tertiary care center with a history of swelling in the left inguinoscrotal region since past 2 years. Swelling has gradually progressed in size over the past 2 years from its initial size of 2 × 2 cm to its present size of 8 × 6 cm. Patient had no history of pain and change in the size of swelling with change in posture (supine or erect position). There is no significant history of trauma. Patient had history of left sided inguinal hernia repair through transabdominal preperitoneal approach three years ago. No significant medical comorbidities were present. There is no significant family or psychosocial history. Thorough clinical examination revealed an elastic firm, nonreducible, nontender swelling in the left inguinoscrotal region measuring 8 × 6 cm with smooth surface and no signs of inflammation. Testis was palpable separately with positive cough impulse. Abdominal examination was insignificant, and other hernial sites were intact. As an adjunct to clinical examination, an ultrasonography of inguinoscrotal region was done, which showed a heteroechoic lesion of size 7 × 6 cm in the left inguinoscrotal region with probable features of lipoma or desmoid. In view of persisting diagnostic dilemma, a fine needle aspiration cytology from the swelling was done which suggested the features of fat containing benign lesion probably lipoma. Surgical exploration of the swelling was conducted by a team of surgeon headed by an additional professor of surgery with 12 years of surgical experience. Intraoperative findings revealed that tumor is originating from the spermatic cord (Figure 1). The excised tumor was of size 7 × 6 × 5 cm (Figure 2). This tumor was firm, greyish white pedunculated globular with smooth surface and regular margins (Figure 3). Cord and cord structures were separated from the swelling, and adequate hemostasis was achieved. The testes were free from the swelling, and no hernial sac was identified. Histopathological examination of the lesion revealed lobules of mature adipocytes within the stroma of spermatic cord (Figure 4(a)) and lobules of mature fat forming mass within cord tissue, separated by fibrous septae (Figure 4(b)). Postoperative period was uneventful, and the patient was discharged on day five. Regular follow-up of the patient was done at 1 month, 3 months, and 6 months with no complains.

## 3. Discussion

Beauty of surgery lies in the uncertainties of diagnosis and dilemmas sorted till intraoperative findings and histopathological confirmation. Among all intrascrotal tumor, primary paratesticular tumor constitutes only 7 to 10% [1]. In adults, 75% of these paratesticular tumors originate from the spermatic cord. Swelling in inguinal region with positive cough impulse is generally considered to be a hernia unless proven otherwise especially in a patient with previous history of laparoscopic inguinal hernia repair. Inguinal hernia is more of a clinical diagnosis and demands minimal diagnostic modality for the stipulation of the diagnosis. Although thorough clinical examination leads to the diagnosis in majority of cases, but few instances with features of a firm, nonreducible mass with a positive cough impulse may warrant ultrasonography as in our case which suggested heteroechoic lesion with a possibility of lipoma or desmoid. Further investigations with fine needle aspiration led to the diagnosis of a benign lipomatous swelling. Intraoperative findings in contrary to our clinical and pathological diagnosis demonstrated a well-encapsulated grayish white, firm, and pedunculated mass arising from the spermatic cord which further provide insight for the reconsideration of our diagnosis. Surprisingly, final histopathological report turned out to be a fibro lipoma. Fibro lipoma arising from the spermatic cord is an infrequent entity with very few cases reported in literature. As per our literature review, the first ever case of scrotal fibro lipoma was reported by Huben et al. [3] in a 68-year-old male with the presentation of massive scrotal swelling for the past 2 years; it was of a size 20 × 25 × 30 cm and was confirmed histologically with features of mature fibrous and adipose tissue. Later on, Hegele et al. [4] reported the first case of spermatic cord fibro lipoma in a 55-year-old patient with presentation of inguinal swelling and final gross size of 4 × 3 × 2 cm with similar histological features. Tarda et al. [5] reported the second case of spermatic cord fibro lipoma in a 68-year-old patient with tumor size of 13 × 10 × 9 cm with histological features of fibro lipoma and focal myxoid degeneration. We present the third case of spermatic cord fibro lipoma and first of its kind as it was encountered in a 30-year-old young male patient with atypical presentation mimicking as inguinal hernia which further mystified the case. It was finally confirmed on histopathology with features of encapsulated mass line with a fibro collagenous tissue and composed of mature adipose tissue with macrophages, calcifications, and areas of fat necrosis indicative of fibro lipoma with secondary fat lysis. It is of paramount importance to rule out liposarcomatous pathology in cases of fibro lipoma. In index case, malignancy was not suspected in consideration to young age of the patient and absence of liposarcomatous changes such as high mitotic activity, invasion, and cellular atypia.

## 4. Conclusion

Spermatic cord fibro lipoma is rare kind of paratesticular tumor. Atypical presentation creates diagnostic dilemma and may lead to intraoperative astonishment. Surgical excision of the tumor after preoperative evaluation is the treatment of choice. Although it is reported to effect older individuals, but it may be encountered in younger population as well.

## Additional Points

*Guarantor*. Dr. Anil Kumar is an Additional Professor in Department of General Surgery (Trauma and Emergency), All India Institute of Medical Sciences, Patna, India (mobile: 91 9835699103, email: dranil4@gmail.com).
